# Reprogramming Tumor-Immune Cell Interface in Solid and Hematological Malignancies to Enhance Response to Therapy

**DOI:** 10.1186/s13046-018-0710-x

**Published:** 2018-03-05

**Authors:** Maria Teresa Di Martino, Francesca Zazzeroni, Massimo Donadelli, Claudia Chiodoni, Michele Caraglia, Katia Scotlandi, Stefania Meschini, Carlo Leonetti

**Affiliations:** 1 Dept. of Experimental and Clinical Medicine, University of Catanzaro “Magna Graecia”, Catanzaro, Italy; 2 Dept. of Biotechnological and Applied Clinical Sciences, University of L’Aquila, L’Aquila, Italy; 3 Dept. of Neurosciences, Biomedicine and Movement Sciences, Section of Biochemistry, University of Verona, Verona, Italy; 4 Molecular Immunology Unit, Dept. of Research, Fondazione IRCCS Istituto Nazionale dei Tumori, Milan, Italy; 5 Department of Biochemistry, Biophysics and General Pathology, University of Campania “L. Vanvitelli”, Naples, Italy; 6 Experimental Oncology Lab, CRS Development of Biomolecular Therapies, Orthopaedic Rizzoli Institute, Bologna, Italy; 7 National Center for Drug Research and Evaluation, National Institute of Health, Rome, Italy; 8 UOSD SAFU, Regina Elena National Cancer Institute, Rome, Italy

## Presentation of the conference

The 30^th^Annual Conference of Italian Association of Cell Cultures (AICC) was held at Fondazione IRCCS Istituto Nazionale dei Tumori, in Milan, on November 27–28, 2017, with the Scientific Coordination of Claudia Chiodoni and Mario Paolo Colombo, from the same Institute. The conference was opened by Katia Scotlandi, President of the Italian Association of Cell Cultures. The main objective of the conference was to discuss the present knowledge on the role of immune cells in the tumor microenvironment, shaped by cells promoting tumor survival that could be reeducated to treat cancer. The meeting was organized in two days with 5 thematic sessions, an opening and a closing lecture, with national and international speakers. The organizers truly thank those who took part in the conference and made the Conference a success.

## Opening lecture

**Fig. 1:**
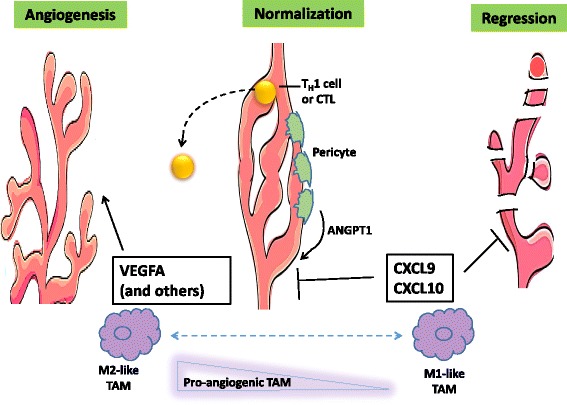
Adapted from De Palma et al. [19]. Manipulation of chemokine-chemokine receptor signaling pathways could reshape the immune and biological phenotypes of a tumor in a manner that increases effectiveness of immunotherapy

Michele De Palma, from École Polytechnique Fédérale de Lausanne, Swiss Institute for Experimental Cancer Research, in Lausanne, Switzerland, opened the 30th Annual Conference of AICC with the lecture “Reprogramming the tumor immune microenvironment”. His lecture revised the role of the tumor microenvironment (TME) in the regulation of angiogenesis highlighting the potential vulnerabilities that could be targeted to improve the efficacy of anti-angiogenic cancer therapies (Fig. 1).The TME represents the primary location where tumor cells and the host immune system interact. Through interactions between chemokines and chemokine receptors, different immune cells are recruited into the TME where they influence tumor progression and therapeutic outcome. De Palma reported important findings suggesting that the manipulation of chemokine-chemokine receptor signaling pathways could reshape the immune and biological phenotypes of a tumor in a manner that increases effectiveness of immunotherapy. Based on recent findings, it is predicted that targeting both pro-tumor and anti-tumor chemokine-chemokine receptor signaling pathways, in combination with other immunotherapies, such as programmed cell death-protein 1 (PD-1) and ligand 1 (PD-L1) blockade, could give clinical benefit in cancer patients. In this context, De Palma addressed the extrinsic regulation of angiogenesis by TME. Pathological angiogenesis is a hallmark of cancer and a therapeutic target. The angiogenic programming of a tumor tissue is a process regulated by many players  such as tumor cells together with different tumor-associated stromal cells and their bioactive products, including cytokines/chemokines or growth factors, the extracellular matrix and secreted microvesicles. De Palma and coworkers demonstrated that the combination of angiopoietin-2 (ANGPT2) and vascular endothelial growth factor A (VEGFA) blockade by a bi-specific antibody (A2V) provided therapeutic benefits, as compared to the single agents, in both genetically-engineered and transplantable tumor models, including metastatic breast cancer, pancreatic neuroendocrine tumor and melanoma. He reported that A2V promoted vascular regression, tumor necrosis, and antigen presentation by intratumoral phagocytes. A2V also normalized the remaining blood vessels and facilitated the extravasation and perivascular accumulation of activated, interferon-γ (IFNγ)–expressing CD8+ cytotoxic T lymphocytes (CTLs). Whereas the anti-cancer activity of A2V was, at least in part, CTL-dependent, perivascular T cells concurrently up-regulated the expression of the immune checkpoint PD-L1 in tumor endothelial cells. IFNγ neutralization blunted this adaptive response, and PD-1 blockade improved tumor control by A2V in different cancer models. He concluded that immune cells could be considered as key effectors of antiangiogenic therapy and support the rationale for co-targeting angiogenesis and immune checkpoints in cancer therapy [1]. De Palma also reported their recent results on the role of microRNA (miRNA) in reprogramming tumor-associated macrophages (TAMs). He showed their recent findings that myeloid-specific inactivation of the miRNA-processing enzyme, DICER, promotes the functional polarization of TAMs to a M1-like phenotype characterized by hyperactive IFN-γ/STAT1 signaling. This rewiring decreased the immunosuppressive capacity of TAMs and favored the recruitment of activated CTLs to the tumors. CTL-derived IFN-γ exacerbated M1 polarization of Dicer1-deficient TAMs and inhibited tumor growth. Surprisingly, DICER deficiency in TAMs abrogated the anti-tumor effects of macrophage depletion by anti-CSF1R antibodies, and allowed complete tumor eradication by PD-1 checkpoint blockade or CD40 agonistic antibodies. Moreover, the group of De Palma observed that genetic rescue of Let-7 miRNA activity in Dicer1-deficient TAMs partially restored their M2-like phenotype and reduced tumor-infiltrating CTLs. The findings presented by De Palma indicated that DICER/Let-7 activity opposes IFN-γ-induced, immunostimulatory M1-like TAM activation, with potential therapeutic implications and suggested that, the inhibition of Let-7 activity may provide a strategy for reprogramming immunosuppressive TAMs into cells capable of stimulating anti-tumor immunity. All together the provocative findings presented by De Palma support the hypothesis that the reprogramming of TAMs to an immunostimulatory mode can synergize with several anti-cancer therapies that, either directly or indirectly, may enhance the endogenous immune response against the tumor [2].

## Session 1

### 3D cultures and microfluidics: New tools for modeling tumor—Stroma interaction

In vitro bi-dimensional (2D) cell culture models have been extensively used to investigate tumor cell behaviors and to assess cancer cell responsiveness to therapies. However, 2D cell cultures represent an over-simplified system due to the absence of the specific tumor microenvironment context, known to be critically essential in cancer progression. In order to overcome these limitations of 2D cell models, three-dimensional (3D) cultures and microfluidic systems have been recently developed as “closer-to-in vivo” systems in which three-dimensionality, structural organization and presence of flow are accomplished. For the 3D cultures, a critical step is the choice of matrices and scaffolds. Several types of matrices and scaffolds of both organic and inorganic nature are being currently employed; the most commonly used are agarose, collagen, fibronectin, gelatin, laminin, and vitronectin. The choice of such matrices and scaffolds is mainly based on the cell type and the specific micro-architecture needed to be obtained.

In this context, Serena Danti from University of Pisa (Italy) clearly demonstrated that the architectural features of the matrices influence scaffold cell colonization, cell morphology, growth and activity. 3D cancer models of pancreatic ductal adenocarcinoma (PDAC) were interfaced with different polymeric scaffolds. Fabrication of both sponge-like matrices and fiber mesh scaffolds with different internal architectures were obtained by using two biocompatible polymeric formulations – poly(vinyl alcohol)/gelatin (PVA/G) mixture and poly(ethylene oxide terephthalate)poly(butylene terephthalate (PEOT/PBT) copolymer – and different conventional scaffolding techniques. Although primary PDAC cells were able to adhere to the inner surface of both spongy-like and fiber scaffolds and the cell viability did not significantly differ among the different matrices, a duct-like morpho structure resembling the local advanced PDAC tissue was obtained only when using spongy-like scaffolds. Indeed, spongy-scaffolds enhanced also production of MMP-2, demonstrating that these types of scaffolds supported the generation of pancreatic tumor models with enhanced aggressiveness [3]. Danti showed also data regarding a 3D in vitro model of HCC based on HepG2 liver cells seeded inside poly(vinyl alcohol) and gelatin (PVA/G) hydrogels and cultured for up to 24 days. In this 3D setting, HepG2 cells were viable for long term and formed aggregates with different morphological features and cytoskeletal organization according to their zonal localization, thus demonstrating that this 3D HCC model could represent a useful tool for investigating mechanisms of tumor progression and invasion.

A relevant aspect of tumor invasion is represented by circulatory patterns and microarchitecture of capillary vessels. Recent advances in the generation of microvascularized systems through microfluidic approaches were nicely presented by Chiara Arrigoni, from IRCCS Istituto Ortopedico Galeazzi of Milan (Italy). She described a microfluidic 3D model to assess transmigration of breast cancer cells (BCC) through micro-vessels followed by colonization of a bone-like microenvironment, which was made of osteo-differentiated BMSCs and hydrogel [4]. The method was based upon polydimethylsiloxane (PDMS) molds, needed to generate complex 3D constructs in which several cell types and biomaterials were incorporated with a high spatial control. Interfaces between gels and culture medium channels were obtained by means of PDMS microposts, allowing the formation of microvascular networks accessible from the medium channel itself. Moreover, the most organized vascular network was achieved supplementing co-culture of mesenchymal and endothelial cells with ANG-1 and VEGF. These studies clearly indicate that microfluidic technologies dramatically improve the significance of in vitro 3D biological models, and represent promising tools to develop in vitro 3D models in which the microenvironment and cell distribution can be spatially controlled and biophysical and biochemical features can be modulated.

## Session 2

### Tumor metabolism

**Fig. 2:**
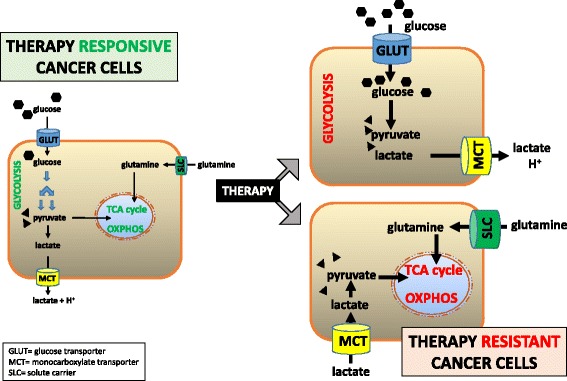
Cancer therapy can strongly influence the metabolic reprogramming of resistant cancer cell subpopulations. They can rewire their metabolism towards glycolysis or OXPHOS on the basis of the type of cancer therapy

Metabolic deregulation is an established hallmark of cancer cells. Also in immune cells, dramatic metabolic changes are required to effectively achieve the various cellular functions that create a dynamic immune response against invading pathogens and tumor cells. Recent studies have provided mechanistic details of the contribution of the aberrant modulation of cancer metabolism in the regulation of tumor growth and response to chemotherapy, radiotherapy and/or immunotherapy. It is well recognized that most cancer cells enhance glucose and glutamine consumption to satisfy their energy demand and biosynthesis requirements for fast proliferation. Importantly, Warburg stated that, even in the presence of oxygen, cancer cells show increased glycolysis using only a small fraction of glucose for oxidative phosphorylation (OXPHOS) [5]. More recently, it has been reported in different tumors types that the energetic metabolism of resistant cell subpopulations is either Warburg-like or OXPHOS-addicted, with conceivable explanation as to which metabolic phenotype could be advantageous during tumor development and adaptation to therapy. Some studies collectively suggest that the type of therapy can strongly influence the metabolic reprogramming of resistant cancer cells. Therefore, therapy may have an active role in selecting resistant clones and, depending on the mechanisms of action, drugs might confer a high degree of plasticity to the cancer cells, making them particularly clever in rewiring their metabolic network (Fig. 2).

In this context, Stefano Indraccolo, from Istituto Oncologico Veneto (Padua, Italy) clearly elucidated the metabolic rewiring in tumors induced by anti-angiogenic therapy, such as VEGF ligand-blocking drugs or drugs blocking VEGFR signaling [6]. It is recognized that anti-angiogenic therapy causes a rise in hypoxia in the targeted tissues and subsequent HIF1α-mediated up-regulation of several glycolysis genes, including glucose transporter 1 (GLUT1), lactate dehydrogenase A (LDHA), and MCT4, a lactate transporter that is often over-expressed by cells surrounding necrotic areas in solid cancers. In addition, HIF-1 triggers the pyruvate dehydrogenase kinases (PDKs), which deactivate the mitochondrial pyruvate dehydrogenase complex and thereby decrease the flow of glucose-derived pyruvate into the mitochondria. These data support the hypothesis that anti-angiogenic drugs might rewire tumor metabolism and promote the Warburg effect. Beyond HIF-1, the comprehensive view of metabolic alterations caused by anti-angiogenic drugs in tumors might lead to the discovery of novel predictive biomarkers of response. For instance, tumors bearing genetic defects in LKB1, a kinase stimulating AMPK signaling, might undergo metabolic crash following anti-angiogenic therapy by VEGF blockade. This hypothesis is currently explored in lung cancer, as LKB1 inactivation is relatively common in this malignancy. Furthermore, some studies underline the possible existence of intra-tumor metabolic heterogeneity not only forced by local pathophysiological conditions, such as hypoxia or glucose deprivation, or adaptation to a specific environment, but rather intrinsic to tumor cells. Indraccolo concluded that this multifaceted metabolic heterogeneity is still poorly described and it might be connected with the genetic heterogeneity of solid tumors, given the proven link between certain oncogene/tumor suppressors and metabolism (such as Akt/KRAS/glycolysis and MYC/glutaminolysis), or, alternatively, be accounted for by epigenetic modifications.

Recent discoveries also indicate that an important Achilles’ heel of many types of cancer cells is their incapacity to adapt to starvation. While healthy cells respond to nutrient and growth factor deficiency by activating maintenance and stress response mechanisms that make them more resistant to diverse types of injuries, including chemotherapy, this type of reaction is frequently compromised in cancer cells, mainly as a consequence of aberrant oncogene activation. Following this view, Alessio Nencioni, from University of Genova (Italy), outlined that starvation conditions rise the ability of tyrosine kinase inhibitors (TKIs), as erlotinib, gefitinib, lapatinib, crizotinib and regorafenib, to arrest cancer cell growth, to inhibit MAPK signaling and to reinforce E2F-dependent transcription inhibition. In cancer xenograft models, both TKIs and cycles of fasting slowed tumor growth, when associated, were significantly more effective than either type of treatment alone. He concluded that cycles of fasting or of specifically designed fasting-mimicking diets should be considered in clinical studies as a mean to enhance the action of standard therapies [7].

Since neoplastic cell growth needs a constant supply of lipids with balanced molecular composition to create new cell membranes, a process dependent on de novo synthesized fatty acids (FAs), Alessandra Carè, from Istituto Superiore di Sanità in Rome, studied the involvement of stearoyl-CoA desaturases (SCDs) in tumor progression [8]. SCDs are endoplasmic reticulum enzymes that catalyze the introduction of the first double bond in the cs-Δ9-position to convert saturated FAs (SFAs) into mono-unsaturated FAs (MUFAs). In particular, she showed that SCD5 is down-regulated in advanced melanoma and its restored expression significantly restricted melanoma malignancy, both in vitro and in vivo, through a mechanism regulating the secretion of extracellular matrix proteins, such as secreted protein acidic and rich in cysteine (SPARC) and collagen IV and of their proteases, such as cathepsin B. Exogenous over-expression of SCD5 or supplementation of its enzymatic product, oleic acid, diminished the intracellular pH and, in turn, the vesicular trafficking across plasma membranes, along with the spreading of melanoma. This intracellular acidification is, at least partially, due to SCD5-induced reduction of the vacuolar H^+^-ATPase, a proton pump whose inhibition changes the secretion profile of cancer cells. In addition, she demonstrated the existence of a negative feedback loop between SCD5 and miR-221&222, in good agreement with their opposite functions and with the less invasive more differentiated phenotype, associated with SCD5 re-expression in advanced melanomas. These data support a role for SCD5 and its enzymatic product, oleic acid, in the defense against malignancy, offering molecular details for the beneficial Mediterranean diet.

## Session 3

### Tumor microenvironment in hematological malignancies

In the last decades, it has become clearly evident that components of the tumor microenvironment (TME) are far from being mere bystander elements, but rather actively contribute to tumor development and progression. While this concept is by now a milestone for most solid tumors, the tumor microenvironment in hematological malignancies is less studied.

Indeed, while the understanding of the pathogenesis and progression of lymphomas and other hematological malignancies has considerably advanced, due  to the recent high-throughput technologies, such as next-generation sequencing, leading to the identification of the genetic alterations and cellular pathways involved, the relevance of the cellular context in which these malignancies arise, has been relatively neglected in the past years. Now, it has become clear that, also in hematological tumors, the cross-talk between neoplastic elements and surrounding stromal and immune cells, is critical for tumor progression, providing survival and proliferation signals, as well as protection from the immune attack. Classic Hodgkin Lymphoma (cHL) represents an extreme example of hematological tumor in which the tumor microenvironment is of outmost relevance, being this neoplasia composed by a low number of transformed cells surrounded by normal accessory cells, such as macrophages and monocytes that actively contribute in building an immunosuppressive environment. Beside cHL, many other B cell malignancies, such as CLL (chronic lymphocytic leukemia) and DLBCL (diffuse large B cell lymphoma) rely on support of surrounding stromal and immune cells for their progression and dissemination.

In this context Silvia Deaglio, from the University of Turin (Italy), clearly outlined the need for CLL of a “nursing” niche in lymphoid organs. Indeed, the survival of leukemic cells is highly affected by the interactions with non-leukemic cells in these permissive niches where CLL cells subvert the normal architecture of the lymphoid organs and recruit accessory cells, such as stromal cells, dendritic cells and T lymphocytes, all playing an active role in survival and proliferation of CLL. Recently, several studies highlighted the role for hypoxia in re-shaping tumor microenvironment towards immune suppression. Accordingly, the CLL niche has been found to be a highly hypoxic environment in which the low oxygen tension favors the differentiation of circulating monocytes into nursing cells characterized by the expression of immunosuppressive markers such as IDO, CD206, CD163, IL-10 and IL-6, all molecules contributing to the establishment of an immunosuppressive environment in which T cell response is inhibited and T regulatory cells are expanded. These effects are, at least in part, mediated by the adenosinergic system. In low O2 tension, CLL cells undergo a metabolic adaptation that increases the levels of extracellular ATP, enhancing adenosine production and signaling through adenosine receptors, expressed by CLL and surrounding accessory cells. In line with this, targeting the adenosinergic axis, may revert the effects on cell differentiation and may open the way to specific inhibitors as a new therapeutic strategy in CLL [9].

Beside its cellular components, the TME comprises also a number of molecules that all together form the extracellular matrix (ECM). ECM, once considered a mere scaffold for the tumor to grow in size, is now being appreciated also for its regulatory properties. Claudio Tripodo, from the University of Palermo (Italy), elegantly showed how the ECM molecules actively contribute to define the architecture of the secondary lymphoid organs (SLO) and how changing specific ECM proteins may alter the course of the disease. He showed that the deficiency of the matricellular SPARC, in mice prone to lupus-like autoimmunity (Fas^lpr/lpr^) backcrossed to Sparc-deficient animals, led to a higher severity of autoimmunity that eventually resulted in the development of a CD5+ B cell lymphoma resembling human B-CLL. On the other hand, when Fas^lpr/lpr^mutation was transferred to mice deficient for a different matricellular protein, osteopontin (OPN), mice showed severe and accelerated secondary lymphoid organ hyperplasia that evolved to a different type of lymphoma in aged mice, characterized by an accumulation of a non-canonical B cell population, suggestive of a high-grade DLBCL of the Activated B cell type.

DLBCL is a molecularly heterogeneous disease whose genetics may have clinical implications for prognosis and choice of treatment. However, the limited access to fresh tumor material has hindered the use of DLBCL mutational profiles for clinical practice. In such a context, Davide Rossi from the Institute of Oncology Research in Bellinzona (Switzerland), proposed DLBCL genotyping by analyzing cell-free DNA, which is released into the blood by tumor cells undergoing apoptosis and can be used for the identification of DLBCL mutations, clonal evolution, and mechanisms of resistance. In particular, NGS of plasma cfDNA of DLBCL patients has a high sensitivity and specificity in discovering mutations, even those that are undetectable in the tissue biopsy because harbored in clones anatomically distant from the biopsy site [10].

## Session 4

### Immunotherapy and treatment resistance

The session was dedicated to the optimization of Adoptive T cell therapy (ACT) against cancer and to the strategies developed to overcome the immune resistance of cancer cells. ACT has become a promising immunotherapeutic option for cancer patients. The proof for ACT therapeutic efficacy was first obtained with allogenic T cells and then reproduced with T cells isolated from patients’ tumor samples (i.e. tumor-infiltrating lymphocytes) [11]. It is now clear that specificity of ACT products can be educated by genetically engineering T cells with classical T Cell Receptors (TCR) or chimeric antigen receptors (CAR). Anna Mondino, from San Raffaele Hospital (Milan) described the role played in solid tumors by the stromal barrier and/or interference and by the presence of frequent changes in immunogenic epitopes in the tumor due to genetic instability. These tumor-related mechanisms severely limit ACT-based approaches. It could be possible to overcome these limitations through the concomitant immunogenic targeting of both cancer and stroma cells. This aim can be achieved by “dual targeting” strategies based on combination of TCR/CAR-redirected T cell products and their association with drugs targeting tumor-vessels and/or epigenetic modifiers, with the ability to sensitize tumors to T cell recognition [12]. Existing data have proven synergistic effects in combined settings and suggest that additional benefit can be achieved by additional combinatorial therapeutic approaches in ACT of solid tumors. Andrea Anichini, from Fondazione IRCCS Istituto Nazionale dei Tumori (Milan), has described the down-modulation of T-cell antitumor effector functions by immune inhibitory factors within the tumor microenvironment [13]. Such molecules include cell surface proteins (such as programmed cell death 1 ligands 1 and 2(PD-L1 and PD-L2) also called immune checkpoint inhibitors and cytokines (such as TGF-β and IL-10). Immunomodulatory mAbs can be used to escape from local immunosuppression blocking suppressive receptors (such as, programmed cell-death 1 (PD-1) or cytotoxic T-lymphocyte antigen 4 (CTLA-4). The clinical responses to these mAbs have been relevant in the clinical setting and the results have clearly shown that in some cases they can induce long lasting clinical responses, significantly prolonging the survival of the patients. In addition, some malignancies including malignant melanoma, are very sensitive to this therapeutic strategy, while others such as pancreatic ductal adenocarcinoma are poorly responsive. These clinical evidence have pushed the investigators in searching for biomarkers and molecular factors involved in the resistance to immunotherapy with immune checkpoint-blocking mAbs. Several findings have suggested that in tumors the increased lymphocyte infiltration and the high load of mutations are correlated with clinical responses to immunecheckpoint inhibitors. This implies that the targeting of cancer cells by cognate T cells activated by the mAbs, requires an active HLA class I antigen processing machinery (APM). Therefore, HLA class I APM component defects can play an important role in both innate and acquired resistance to immunotherapy with immune checkpoint-blocking mAbs. The mechanisms determining these defects include alterations in histone acetylation, MAPK pathway activation, and methylation of the promoters of genes encoding HLA class I APM components. Several strategies can be exploited in order to overcome this protective mechanism including the use of histone deacetylase inhibitors, inhibitors of MAPK pathway activation, and demethylating agents. In this light, targeted therapy based on BRAF- and MEK-inhibitors and immunotherapy with immune checkpoint inhibitors currently represents the standard of care as first-line treatment for advanced/metastatic melanoma disease. Such therapies induce a 3-year overall survival rate of 45% and a 5-year overall survival rate of 30%, respectively. Similar strategies have been successfully developed with other target-based agents such as anti-HER2 and anti-ALK. Another way to overcome the resistance to immune checkpoint inhibitors is the combination of different immunotherapy approaches. Beyond the combined use of the PD-1 and CTLA-4 blocking mAbs, the latter mAbs can be also combined with other immunomodulatory molecules, such as anti-LAG3, anti-GITR, anti-IDO, anti-CSF and anti-OX40 Abs. While no increase in side effects was observed, promising clinical responses were recorded in highly pre-treated patients. Finally, Marina Garassino, from the same Institute, illustrated the scenario opened by the use of new immune checkpoint inhibitors showing the role played by PD-L1 status in redirecting the response and the survival of the patients.

## Session 5

### Tumor-associated immunosuppression

Tumor progression is associated with a general state of immune suppression that acts both locally, at the tumor site, and systemically. Several players take part in establishing such immune-suppressive environment, such as myeloid suppressor cells (myeloid-derived suppressor cells/MDSCs and tumor-associated macrophages/TAMs), regulatory T cells, inhibitory cytokines and immune checkpoint receptors, which act in concert with cancer cells to inhibit anti-tumor immunity.

Licia Rivoltini, from the Fondazione IRCCS Istituto Nazionale dei Tumori in Milan, focused her talk on MDSC in melanoma, providing mechanistic evidence for their expansion/regulation and suggesting their feasibility as prognostic markers [14].

She described the development of an in vitro model of MDSC generation, based on monocytes conditioned with melanoma exosomes (EXO-MDSC) and leading to the generation of HLA-DR^low^, TGFβ/IL6/CCL2-secreting immunosuppressive cells. Several miRNAs were strongly regulated in EXO-MDSC, and were responsible for MDSC generation and suppressive phenotype. By in vivo experiments she showed that injection of melanoma exosomes in mice induces accumulation and activation of MDSC. Rivoltini’s research group also identified a myeloid index score (MIS) in the peripheral blood mononuclear cells that defines 4 subsets of metastatic melanoma patients with increasing disease aggressiveness and worse prognosis.

The other major myeloid suppressive subset expanded in tumor conditions, is represented by TAMs that were clearly presented by Antonio Sica [15], from Università del Piemonte Orientale (Novara, Italy). A plethora of evidence indicate that macrophages are characterized by an elevated functional plasticity being able to express different functional programs in response to different microenvironmental signals, rendering the M1-M2 paradigm of macrophage polarization a limited view. Nevertheless, in general, TAMs, during tumor progression, acquire a skewed M2 phenotype and express pro-tumor activities. Studies from Sica’s research group provided evidence that TAMs are characterized by a status of tolerance in response to lipopolysaccharide (LPS) and other proinflammatory signals and this state is dependent on nuclear overexpression of the p50 NF-κB inhibitory homodimer. In agreement, they found delayed tumor progression and increased survival in p50 NF-κB–deficient mice in both melanoma and fibrosarcoma models. More recently his group investigated the role of p50 NF-κB in regulating the phenotype of TAMs in colorectal cancer (CRC). Using an in vivo mouse model of colitis-associated cancer (CAC) and ApcMin mice, they found that p50−/− mice displayed exacerbated colitis but fewer and smaller tumors, and enhanced levels of Th1 cytokines/chemokines, such as IL-12 and CXCL10, suggesting that p50-driven inhibition of M1-polarized gut inflammation is able to support CRC development.

Sabina Sangaletti, from the Fondazione IRCCS Istituto Nazionale dei Tumori in Milan (Italy) addressed the issue of tumor-associated immune-suppression from a different point of view, focusing on the role of the ECM in such a context. Indeed, as already underlined by Claudio Tripodo in the context of hematological malignancies, the ECM exerts regulatory activities that, within the TME, actively contribute to many aspects of tumor progression acting on both tumor and immune cells. In particular, the study of Sangaletti and colleagues showed that ECM regulates tumor-associated immuno-suppression through the modulation of myeloid cell features, from their expansion, localization and functional activities [16]. The important role of ECM in tumor progression is confirmed by its prognostic impact, such as in case of high-grade breast cancers (BC) in which ECM-gene profiles correlate with clinical outcome and response to therapy. Among the different ECM-related signatures, only the ECM3 profile, enriched in SPARC, COL1A1, COL5A2, LAMA4, COL6A3 and MMP11 genes, was shown to identify breast tumors endowed with epithelial mesenchymal transition (EMT) features and poor response to therapy and characterize about 40% of BC. Modeling in mice such tumors, Sangaletti showed that the re-expression of SPARC, the leading gene of the ECM3-signature and a master regulator of collagen deposition, in SPARC-low or deficient BC cells, was sufficient to reproduce the main ECM3 BC profile, including the enrichment in mesenchymal features. In these tumors, the EMT program depended on the ECM-mediated control of MDSC activity and the recruitment of these cells at the tumor site was functionally relevant for preserving the mesenchymal phenotype of ECM3/SPARC-expressing BC. In the same pre-clinical model the inhibition of MDSC suppressive functions, through the administration of Zoledronic acid (ZA), an aminobisphosphonate, reverted the EMT phenotype rendering ECM3/SPARC-expressing BC sensitive to chemotherapy.

The last talk was held by Silvia Piconese, from the Università La Sapienza in Rome, who introduced another component of the tumor immune-suppressive environment, the regulatory T cells (Tregs), a CD4 T cell subset physiologically involved in the suppression of unwanted immunity and inflammation, and pathologically in the inhibition of anti-tumor responses. The data showed by Piconese on human cancers, demonstrate that Tregs accumulate in hepatic tissues affected by premalignant condition (cirrhosis) or hepatocellular carcinoma (HCC) and that OX40, a member of TNF receptor superfamily, is highly expressed by Tregs at the tumor site, and promotes their expansion and stability [17]. Interestinlgy, she addressed the issue of the metabolic routes sustaining the expansion of Tregs in cancer that are still unknown. During an immune response, T cells shift from oxidative to glycolytic metabolism in resting/memory or proliferating conditions, respectively. Treg conversion mostly relies on oxidative metabolism, while the expansion of pre-existing Tregs is dependent on anabolic pathways, in particular cholesterol biosynthesis. In the tumor microenvironment, glucose deprivation, because of its high consumption by neoplastic cells, leads to functional paralysis of anti-tumor effector cells. Therefore, it is likely that, in such hostile microenvironment, Tregs, which are less susceptible to glucose deprivation, may prevail. Using in vitro and in vivo mouse models, her group studied Treg-intrinsic lipid metabolism exploring the idea that Tregs critically rely on a lipo-synthesis-fatty acid oxidation circuitry, for their proliferation and function.

## Closing lecture and remarks

The end of the meeting has been enriched by the brilliant talk of Jürg Schwaller (from University of Basel, Switzerland) who has recently discovered that the cellular origin is a critical determinant in the aggressiveness of acute myeloid leukemia (AML), as depends on the type of precursor cell in which the genetic alteration occurs [18]. In particular, the attention was focused on the leukemia fusion gene which is the result of chromosomal translocation associated to extra-medullary tumor infiltration and frequent relapses in humans. To perform their studies, an inducible transgenic mouse model was generated and it was shown MLL-AF9 expression in long-term hematopoietic stem cells (LT-HSC) invasive and chemo-resistant. In fact, this subtype of cells exhibited clonogenic growth, accompanied by expression of genes involved in migration and invasion in vitro. Moreover, LT-HSC-derived AML cells were highly tumorigenic in vivo and expressed genes related to epithelial-mesenchymal transition (EMT). Transcriptomic analysis led to the identification of several genes, such as Evi1 and Erg, which expression is related to disease aggressiveness; moreover, it was demonstrated that EMT transcription factor ZEB1 controls AML cell migration and invasion. These data were confirmed in patients thus leading to the general significance that these genes could be useful for patient classification and represent potential biomarkers or targets for AML.

In conclusion, the 30^th^ annual conference of Italian Association of Cell Culture (AICC) held at the prestigious cancer institute “Fondazione IRCCS Istituto Nazionale dei Tumori”, has seen more than 100 participants, mostly of them young researchers, who actively participated in discussion. The meeting highlighted the recent advances in the understanding the interplay between stroma component and normal cells in solid and hematological tumors. All presentations, including invited speaker and selected talks, have emphasized that the complex network of tumor cells, immune cells, fibroblasts and endothelium has a key role in affecting important aspects of cancer outcome and could provide key diagnostic and prognostic information. Moreover, the data about mechanisms of activation and resistance, as well as combination approaches, are essential for the establishment of more effective therapeutic options.

Finally, as AICC tradition dictates, young researchers have been awarded with prizes for best posters and best oral presentation, both as an acknowledgement of the scientific quality of their work and to encourage them to continue in finding new concepts for a better cure of cancer.

We would like to thank everyone who attended this AICC Annual Conference.
